# Body Composition, Eicosapentaenoic Acid, and Vitamin D are Associated with Army Combat Fitness Test Performance

**DOI:** 10.1080/15502783.2022.2094717

**Published:** 2022-07-05

**Authors:** Jeffery L. Heileson, Jared M. McGowen, Jose M. Moris, Tomas J. Chapman-Lopez, Ricardo Torres, LesLee K. Funderburk, Jeffrey S. Forsse

**Affiliations:** a Human Performance and Recreation, Baylor University, Department of Health, Waco, TX, USA; b Baylor University, Human Sciences and Design, Waco, TX, USA

**Keywords:** Military, fat-free mass index, omega-3 index, 25-hydroxyvitamin D, sport performance

## Abstract

**Background:**

The Army Combat Fitness Test (ACFT), an updated and newly developed metric to assess combat readiness, may require specialized exercise and nutritional interventions. The purpose of this cross-sectional study was to investigate the relationship between body composition, erythrocyte long-chain omega-3 polyunsaturated fatty acids (LC n-3 PUFA), serum vitamin D (VITD) and ACFT performance.

**Methods:**

Sixty cadets (43 males, 17 females; 20.9 ± 3.8 years; 173.6 ± 10.2 cm; 75.6 ± 13.7 kg) completed the 6-event ACFT (3-repetition maximum trap-bar deadlift [3DL], standing power toss [SPT], hand-release pushups [HRPU], sprint-drag-carry shuttle run [SDC], leg tuck [LTK], or plank [PLK], and 2-mile run [2MR]), body composition analysis via dual-energy x-ray absorptiometry (percent body fat [%BF], lean body mass [LBM], fat-free mass index [FFMI (LBM+bone mineral content)]), and an omega-3 questionnaire. A sub-sample (*n* = 50) completed blood draws for fatty acid (eicosapentaenoic acid [EPA] and docosahexaenoic acid [DHA]) and VITD analysis. Significance was set at *p* < .05.

**Results:**

Lower %BF predicted better performance on all ACFT events (*p* < .05), except the PLK. Higher LBM was predictive of better performance on the 3DL, SPT, and SDC (*p* < .05), but no other events. Adjusted FFMI was positively correlated with the 3DL, SPT, HRPU, SDC, and ACFT scores (*p* < .01 for all). Cadet EPA and DHA dietary intake and omega-3 erythrocyte status was well below established recommendations (25.6 ± 33.9 mg, 58.3 ± 78.1 mg, respectively) and an omega-3 index (O3i = %EPA+%DHA in erythrocytes) of 3.96 ± 1.36%, respectively. EPA was associated with better performance on the 3DL (*r* = 0.280, *p* = .049), SPT (*r* = 0.314, *p* = .027), LTK (*r* = 0.316, *p* = .047), and PLK (*r* = 0.837, *p* = .003). After adjusting for body composition, erythrocyte EPA only remained predictive of PLK scores (*p* = .006). Every 0.1% increase in EPA translated into 5.4 (95% CI: 2.1, 8.8) better PLK score. The O3i or DHA were not associated with any performance variables. Cadets’ average serum VITD status was 38.0 ± 14.9 ng∙ml^−1^. VITD was associated with 3DL (*r* = 0.305, *p* = .031), HRPU (*r* = 0.355, *p* = .011), 2MR (*r* = 0.326, *p* = .021), and total ACFT score (*r* = 0.359, *p* = .011). VITD remained predictive of each event after adjustment for body composition. Every 10 ng∙ml^−1^ increase in VITD was associated with 3-point increase in 3DL, HRPU, 2MR scores, and a 13-point increase in the total ACFT score.

**Conclusions:**

Our data highlight the importance of measures of muscularity, LBM and FFMI, on ACFT performance. Additionally, EPA and VITD status is associated with various strength, power, and muscular and aerobic endurance components of the ACFT. While these results could help professionals better assess and train military personnel, especially since these measures are modifiable through exercise and dietary interventions, they are ultimately hypothesis generating and warrant further exploration.

## 1. Background

Physical fitness assessments are widely used by the military and other service organizations to assess and verify personnel job-task capabilities, optimize physical training and performance, assess general health and well-being, and mitigate injury risk [1]. Since 1980, the Army Physical Fitness Test (APFT) has served as the physical fitness assessment to ensure soldiers are physically capable and prepared to deploy and support combat operations [2]. The test consists of 2-minutes of push-ups and sit-ups and a timed 2-mile run. Push-ups and sit-ups measure upper-body and core muscular endurance, while 2-mile run performance measures aerobic endurance and cardiopulmonary health [3]. However, the APFT does not measure physical domains commonly associated with combat activities. Multiple lines of research have recently concluded that the strongest predictors of performance on common soldier and combat tasks are related to strength, power, agility, and sustained anaerobic capacity [4,5], which are not directly tested by the APFT. The ability to accurately assess and predict combat readiness is a chief concern for the Army and its leaders. To alleviate the disconnect between the APFT and the actual physical demands imposed on soldiers in combat situations, the Army Combat Fitness Test (ACFT) was developed and replaced the APFT in October 2020. The six ACFT events, conducted in order, include a three-repetition maximum trap-bar deadlift (3DL), standing power throw (SPT), hand-release push-ups (HRPU), sprint-drag-carry (SDC), leg tuck (LTK) or Plank (PLK), and a 2-mile run (2MR). Mastering the ACFT may require specialized exercise and nutritional interventions.

It is widely established that specific body composition characteristics are related to performance on various fitness and combat tasks in military personnel [6]. For example, previous research has identified lower body fat percentage (%BF) as a key physical trait associated with improved performance on the APFT [7,8] and other military relevant tasks [8], whereas lean body mass (LBM) is not associated with overall APFT performance. This concept is reflected within the Army body composition standards which are largely focused on %BF. However, in line with the transition from the APFT to the ACFT, body composition recommendations to enhance performance may need to be reevaluated. Harty et al. [9] recently proposed the use of a sex-specific lower-limit threshold fat-free mass index (FFMI), a height-adjusted measure of relative muscle mass, as a field-expedient method to identify military personnel potentially at risk of poor combat task performance. Based on data from 619 athletes, the recommended FFMI lower-limit was 19.2 and 16.9 in males and females (15^th^ percentile), respectively. While these thresholds have yet to be formally tested, Roberts et al. [10] showed that FFMI was significantly associated with ACFT performance. Soldier and combat tasks typically require strength, power, agility, and sustained anaerobic capacity mirrored in the newly developed ACFT. These physical domains are closely aligned with measures of muscularity such as LBM or FFMI; not necessarily fat mass or %BF in isolation.

Nutritional strategies leveraged to promote LBM accretion and strength that could potentially improve ACFT performance are similar to those proposed in athletes and are typically focused on total calorie intake, carbohydrates, and adequate protein [11]. While these variables should be considered, a recent analysis found that soldiers exhibited a higher healthy eating index score than controls, with dairy and total protein foods receiving some of the highest scores [12]. Similarly, Lutz et al. [11] reported that military recruits (*n* = 587) consume a diet adequate in carbohydrate and protein. However, Rittenhouse et al. [12] collected biomarker data and concluded that soldiers (*n* = 531) had remarkably low levels of long-chain omega-3 fatty acids, as measured by the omega-3 index (O3i = % eicosapentaenoic acid [EPA] + % docosahexaenoic acid [DHA] within erythrocytes), and vitamin D (25-hydroxyvitamin D [VITD]), the only two nutrients identified as suboptimal or deficient. A study in collegiate athletes, a population not unlike a military cohort, reported that 97% and 90% of athletes had an O3i and VITD value, respectively, below the optimal performance reference range [13]. Several investigations have reported similar sub-optimal O3i and VITD status in athlete and military populations [14–18]. Additionally, studies monitoring the physiological impact of intense military and athlete training have shown that O3i and VITD status significantly decreases from pre- to post-training [18–20]. Incidentally, blood levels of long-chain omega-3 polyunsaturated fatty acids (LC n-3 PUFA), EPA and DHA, and VITD have been shown to be powerful modulators of skeletal muscle physiology [21,22] and are positively associated with muscular performance in healthy adults and athletes [15,23,24]. This is especially relevant for Army personnel transitioning from an endurance-based test (APFT) to a test that includes other combat-relevant metrics such as strength, power, speed, and agility (ACFT).

Considering this recent transition, we speculate that LBM, FFMI, LC n-3 PUFA, and VITD will be associated with ACFT performance, especially for the strength and power events. If so, this will give healthcare providers, namely dietitians, a targeted nutritional intervention to ensure our soldiers perform at their best. Accordingly, the primary objective of our study was to identify associations between overall and individual event ACFT performance and body composition. Our second objective was to examine the relationship between the O3i, EPA, DHA, VITD, and ACFT performance.

## 2. Methods

### Study Design

A cross-sectional descriptive study was conducted to assess the relationship between ACFT performance and body composition, LC n-3 PUFAs, and VITD. Each participant took the ACFT at their training unit and was administered by trained military personnel in accordance with the procedures and standards outlined by Army guidance to assess physical fitness [25]. The official ACFT document was collected from each cadet or provided by their unit’s training records staff. Briefly, the ACFT consists of six events conducted in order: 1) 3DL, 2) SPT (10-lb ball), 3) 2-min max rep HRPU, 4) 300 m SDC, 5) max rep LTK or max hold PLK, and 6) a timed 2MR. The PLK event was originally developed as an alternate event for the LTK; however, cadets were able to select which scored event to complete. Hence, our cohort consisted of 48 (7 females, 41 males) and 12 (10 females, 2 males) cadets to complete the LTK and PLK events, respectively. The events were scored out of a possible 100 points with a maximum score of 600 points. To pass the ACFT, cadets needed to score at least 60 points on each event.

One week following ACFT completion, participants visited the lab on one occasion. Prior to the lab visit, participants were asked to refrain from exercising the previous 24 hours and arrive to the lab fasted for a minimum 8 hours. Upon arriving to the lab, participants were verbally briefed about the study and signed an informed consent form. They subsequently provided a fasting blood sample, filled out an omega-3 fatty acid food frequency questionnaire (FFQ), and had their body composition analyzed via dual-energy x-ray absorptiometry (DXA).

### Subjects

Participants were recruited primarily from the Army ROTC student population in central Texas; however, other local military personnel were included from active duty, reserve, and national guard. Criteria for participation included: (a) completion of an ACFT within the past 6 weeks, (b) at least 18 years of age, (c) not pregnant and (d) free of any known chronic diseases that could affect performance. This study was approved by Baylor University Institutional Review Board (Project ID: 1,675,892-3) and was performed in accordance with the ethical standards of the Declaration of Helsinki. All participants were informed of the experimental procedures and associated risks before providing written informed consent.

### Procedures

*Body Composition Assessments*. Participants body weight (body mass [BM]) was measured (to the nearest 0.1 kg) with a calibrated digital weighing scale and their height was measured to the nearest 0.1 cm with a stadiometer (Seca 703, Hamburg, Germany). Body composition (fat- and bone-free mass [lean body mass (LBM)], fat mass, body fat (%), bone mineral content [BMC] and bone mineral density) was determined using a DXA (Horizon DXA™, Hologic®, Bedford, MA). Prior to the scan, a standard calibration block of a spine was used to calibrate the DXA daily. Also prior to body composition assessment, participants were asked to void their bladder and remove all forms of metal. Additionally, bioelectrical impedance analysis (Tanita, SC-331S, Arlington, IL, USA) was used to determine total body water. Participants were positioned according to the National Health and Nutrition Examination Survey (NHANES) recommendations [26]. To promote and maintain consistency of scan analysis, one researcher analyzed all DXA scans using the Hologic APEX software (version 4.6).

The FFMI was calculated by FFM (LBM+BMC [kg]) divided by height (m) squared [27]. In order to account for the greater muscularity in taller individuals, the FFMI values were adjusted based on previous investigations in which FFMI was regressed against height separately for male and females [27,28]. The slope of the regression line was used to adjust FFMI for males and females. Although height may not affect muscularity as much in females [28], we opted, for consistency, to adjust FFMI similarly for both sexes. We calculated the FFMI_adj_ lower-limit threshold (15^th^ percentile) for all cadets similar to other investigations [27,28]. As recommended by Harty et al. [9], sex-specific thresholds were calculated as a method to screen military personnel for combat readiness and identify those that may be at greater risk of injury.

*Omega-3 Food Frequency Questionnaire*. To determine the cadet’s typical omega-3 intake, an athlete validated 21-item FFQ was utilized [14,15]. The FFQ quantifies the average daily, weekly, or monthly dietary and supplemental intake of marine (EPA and DHA) and plant (alpha-linolenic acid [ALA]) sources of omega-3 fatty acids over the last six months. Results from the FFQ were analyzed based on the methodology and food lists outlined previously [29]. Daily intake of EPA and DHA will be compared to the established 500 mg∙d^−1^ of EPA+DHA recommendations by the Academy of Nutrition and Dietetics [30].

*Blood Sampling and Biochemical Analysis*. Blood samples were taken between 6:00 and 7:30 A.M. following an overnight fast (≥8 hours) and ≥24 hours after exercise. Approximately 21 ml of blood was obtained from the antecubital vein via red top and ethylenediaminetetraacetic acid (K_2_ EDTA) tubes. The red top tubes were allowed to clot in a vertical position at room temperature for 20 minutes prior to centrifuge. All samples were then centrifuged at 3200 rpms for at least 15 minutes, transferred to pre-labeled de-identified polypropylene storage tubes, and immediately stored at −80^◦^C for analysis. Total serum 25-OH VITD concentrations were measured using an enzyme-linked immunosorbent assay (ELISA) kit (Crystal Chem^TM^, Catalog # 80987, Elk Grove Village, IL, USA). The limit of detection for the assay was 5.5 ng∙mL^−1^. Duplicates were run with a mean coefficient of variation of < 7%. Since most of our data are from a single season (fall, n = 50), we did not distribute VITD data based on season. Reference ranges for serum VITD concentrations were: deficient, <20 ng∙ml^−1^; insufficient, 20-29 ng∙ml^−1^; adequate, 30-39 ng∙ml^−1^; optimal ≥40 ng∙ml^−1^ [31].

Packed red blood cells obtained from the K_2_EDTA tubes and transferred to polypropylene storage tubes were shipped overnight on dry ice to a third-party for fatty acid analysis (OmegaQuant, Sioux Falls, SD, USA). Reference ranges for O3i were: sub-optimal, <4%; adequate, 4–8%; optimal, >8% and based on well-established cardiovascular risk categories [32].

### Statistical Analyses

SPSS for Windows version 28 software (IBM, Armonk, NY) was used to analyze the data from this study. Unless otherwise indicated, data are presented as mean ± SD. Data normality was evaluated with histograms and P-P plots and the independence of observations was confirmed via the Durbin-Watson statistic. No outliers were identified.

To determine whether a significant relationship exists between body composition and ACFT performance, a linear regression of LBM and %BF (independent variables) vs the total ACFT score and seven separate scored events (dependent variables) was used. Pearson correlations were used to examine the relationship between FFMI, O3i, red blood cell (RBC) EPA, and DHA, and serum VITD (independent variables) and ACFT performance (dependent variable). For significant Pearson correlations, a hierarchical multiple linear regression was used to account for possible covariates. Specifically, for the strength and power (e.g. 3DL, SPT), LBM was used as a covariate, while %BF was used as a covariate for endurance events (e.g. 2MR). Other independent variables such as age, ethnicity, and sun exposure have been previously identified to be associated with VITD and/or LC n-3 PUFAs; however, the sample characteristics preclude the need to adjust for these variables. Correlation coefficients were interpreted in accordance with guidelines by Cohen [33] as: small (*r* = 0.1-0.29), medium/moderate (*r* = 0.3-0.49), or large (*r* ≥ 0.5). Independent *t* tests were used to evaluate sex differences regarding biomarker data and FFMI categories (<15^th^ compared to >15^th^ percentile) on performance outcomes. Effect sizes for t-tests were calculated as Hedges’ *g* where appropriate based on Lakens [34]. Significance was set *a priori* at *p* < .05. Since our study only included 17 females, we analyzed the data as a whole, which reflects the percentages of men and women in the Army.

## 3. RESULTS

In total, 58 ROTC cadets and 2 other military personnel (43 males, 17 females) volunteered to participate in the study. Due to the low number of other military personnel, the cohort will be referred to as cadets throughout. All participants took the ACFT and, within 7-days, provided data on body composition (*n* = 60) and omega-3 dietary intake; however, a sub-sample opted for a blood draw; hence, LC n-3 PUFA and VITD data were available for 50 cadets. Cadet descriptive data are shown in Table 1.

**Table 1.** Cadet descriptive data.

	Total (n = 60)	Male (n = 43)	Female (n = 17)
Age (years)	20.9 ± 3.8	20.9 ± 4.2	20.7 ± 2.7
Height (cm)	173.6 ± 10.2	177.6 ± 8.3	163.4 ± 7.0
Weight (kg)	75.6 ± 13.7	81.1 ± 11.7	61.9 ± 7.2
BMI (kg∙m^−2^)	25.0 ± 2.9	25.7 ± 3.0	23.1 ± 1.7
Body Fat (%)	17.4 ± 5.2	15.1 ± 3.7	23.3 ± 3.9
Adjusted FFMI	20.8 ± 2.6	21.9 ± 2.0	17.9 ± 1.5
Total Body Water (%)	59.0 ± 4.2	60.4 ± 3.6	55.8 ± 3.8
***ACFT Scores***			
3-RM Deadlift	81.5 ± 14.6	88.5 ± 10.3	63.8 ± 6.8
Standing Power Toss	73.4 ± 16.1	80.4 ± 9.9	55.7 ± 15.3
Hand-Release Pushup	81.8 ± 11.0	85.4 ± 9.4	72.8 ± 9.4
Sprint-Drag-Carry	83.7 ± 20.8	92.9 ± 9.0	60.5 ± 23.9
Leg Tuck	82.7 ± 13.8	84.9 ± 12.6	69.3 ± 13.9
Plank	65.4 ± 12.9	80.0 ± 28.3	62.5 ± 7.6
2 Mile Run	83.4 ± 17.7	88.1 ± 17.6	71.3 ± 11.3
Total ACFT Score	482.6 ± 80.8	520.7 ± 48.3	386.2 ± 64.6

Abbreviations: FFMI, fat-free mass index; ACFT, Army Combat Fitness Test; RM, repetition maximum.

Notes: Data are mean ± SD, rounded to the nearest 0.1. ACFT event scores ≥ 60 are passing.

### Body Composition

Body fat percentage was strongly and negatively correlated to all ACFT events (3DL [*r* = −0.696, *p* < .001], SPT [*r* = −0.631, *p* < .001], HRPU [*r* = −0.686, *p* < .001], SDC [*r* = −0.704, *p* < .001], LTK [*r* = −0.774, *p* < .001], 2MR [*r* = −0.710, *p* < .001], overall ACFT [*r* = −0.845, *p* < .001]), except the PLK (*r* = −.565, *p* = .056). Lean body mass was strongly and positively correlated with 3DL (*r* = .767, *p* < .001), SPT (*r* = 0.777, *p* < .001), HRPU (*r* = 0.283, *p* < .001), SDC (*r* = 0.642, *p* < .001), and overall ACFT performance (*r* = 0.639, *p* < .001). LBM was not correlated with LTK (*r* = 0.283, *p* = .052), PLK (*r* = 0.133, *p* = .679), or 2MR (*r* = .218, *p* = .094) scores. Based on linear regression analysis for body composition, lower %BF was significantly predictive of higher 3DL, SPT, HRPU, SDC, LTK, 2MR, and overall ACFT scores (Table 2). For every 1% increase in body fat, ACFT scores decrease by ~11 points. Body fat percentage was not predictive of PLK scores. After accounting for %BF, higher LBM was associated with higher total ACFT score, 3DL, SPT, and SDC scores. For every 1 kg increase in LBM, ACFT score increases by ~2 points. LBM was no longer associated with HRPU scores.

**Table 2.** Linear regression results for body fat percentage (%BF) and lean body mass (LBM) on ACFT performance outcomes.

	Total ACFT Score	3DL	SPT	HRPU	SDC	LTK	PLK	2MR
Linear Regression								
R^2^	0.78	0.72	0.68	0.48	0.61	0.61	0.32	0.53
*p*	< .001	< .001	< .001	< .001	< .001	< .001	.172	< .001
BF (%)								
B	−10.81	−1.17	−1.00	−1.51	−2.02	−2.80	−1.50	−2.70
SE	1.11	0.23	0.26	0.23	0.38	0.36	0.74	0.35
*P*	< .001	< .001	< .001	< .001	< .001	< .001	.074	< .001
LBM (kg)								
B	1.95	0.68	0.82	−0.07	0.67	0.08	−0.07	−0.26
SE	0.48	0.10	0.12	0.10	0.16	0.14	0.25	0.15
*p*	< .001	< .001	< .001	.491	< .001	.587	.802	.095

Abbreviations: ACFT, Army Combat Fitness Test; 3DL, 3-repetition maximum deadlift; SPT, standing power throw; HRPU, hand-release pushup; SDC, 300 m sprint-drag-carry shuttle run; LTK, leg tuck; PLK, plank; 2MR, 2-mile run

**Figure 1.**
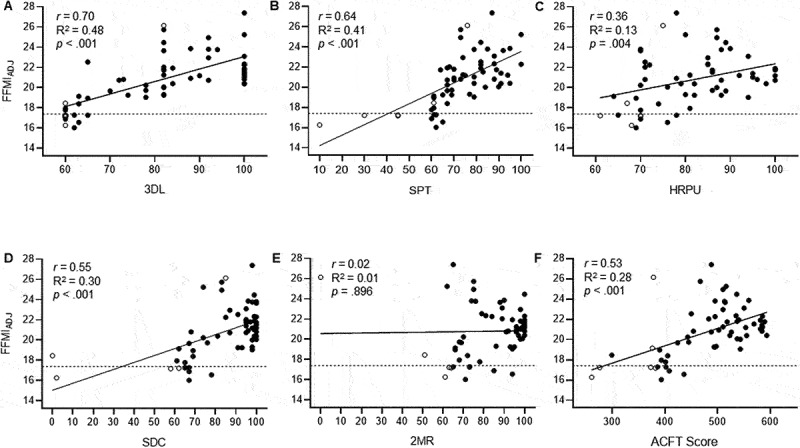
The relationship between adjusted FFMI and Army Combat Fitness Test (ACFT) performance scores on the following events: A. 3-repetition maximum deadlift (3DL), B. standing power toss (SPT), C. hand-release pushups (HRPU), D. sprint-drag-carry shuttle run (SDC), E. 2-mile run (2MR), and F. overall ACFT scores. The dotted line is the delineates the FFMI lower-limit (15^th^ percentile, 17.4) based on the whole cohort. ○ = cadets that failed the ACFT, ● = cadets that passed the ACFT.

Correlations with ACFT performance (total score, 3DL, SPT, HRPU, SDC, 2MR) and FFMI_adj_ are shown in Figure 1A-F. There was a significant positive and strong correlation between FFMI_adj_ and total ACFT score, 3DL, SPT, and SDC and a moderate correlation with HRPU. FFMI_adj_ was not correlated with performance on the LTK (*r* = 0.24, *p* = .102) orPLK (*r* = 0.38, *p* = .225). The 15^th^ percentile for FFMI_adj_ was 17.4 (16.5 and 20.1 for females and males, respectively) for all cadets. Of the six cadets that failed the ACFT, four (66.7%) of them had an FFMI_adj_ < 17.4, whereas the remaining two cadets (3.9%) had an FFMI_adj_ > 17.4. Table 3 reports the ACFT scores based on FFMI status. When using sex-specific 15^th^ percentile FFMI_adj_ cutoffs, only one cadet below the lower-limit threshold failed the ACFT. The male and female cadets identified as having a FFMI_adj_ < 15^th^ percentile scored significantly lower on the 3DL (72.6 vs 83.1, *p* = .045), SPT (64.2 vs 75.0, *p* = .031) and LTK (73.8 vs 84.4, *p* = 0.044) events. There were no performance differences on the HRPU (*p* = .917), PLK (*p* = .682), or 2MR (*p* = .836) events or overall ACFT scores (*p* = .251).

**Table 3.** Army Combat Fitness Test (ACFT) scores based on fat-free mass index (FFMI) categories.

	FFMI Categories			
	>15^th^ Percentile (n = 51)	<15^th^ Percentile (n = 9)	*p*-value	Effect Size
3-RM Deadlift	85.1 ± 12.8	61.3 ± 1.8	< .001	1.86
Standing Power Throw	77.7 ± 11.1	**48.9 ± 18.6**	< .001	2.32
Hand-Release Pushup	83.9 ± 10.3	69.7 ± 5.1	< .001	1.46
Sprint-Drag-Carry	88.1 ± 17.4	**59.0 ± 22.0**	< .001	1.61
Leg Tuck*	84.0 ± 13.1	62.3 ± 2.5	.007	1.69
Plank*	66.7 ± 16.3	64.2 ± 9.7	.754	0.19
2-Mile Run	85.3 ± 18.0	72.1 ± 11.0	.038	0.77
Total ACFT Score	502.7 ± 66.2	368.6 ± 59.6	< .001	2.05

Notes: Data are mean ± SD. **Bold** indicates average ACFT scores that are below retention standards (failed event, < 60 points). RM, repetition maximum. Effect sizes were calculated as Hedges’ *g* according to Lakens [34]. *Cadets had the option to complete the leg tuck or plank, as such the sample sizes were different: Leg tuck, n = 45 and 3 for > 15^th^ and < 15^th^, respectively; Plank, n = 6 and 6 for > 15^th^ and < 15^th^, respectively.

### Dietary Omega-3 Polyunsaturated Fatty Acid Intake

The average daily dietary intake of EPA, DHA, and ALA was 25.6 mg ± 33.9, 58.3 mg ± 78.1, and 158.5 mg ± 325.9, respectively, for all cadets. No cadet reported an EPA+DHA intake ≥ 500 mg∙d^−1^. Of 60 cadets, only 15% (n = 9) consumed the dietary recommended 2 servings of fish per week. Cadets meeting the dietary recommendation for fish intake had an average O3i > 4%. Cadets that reported no intake of fish, less than once per month, and once per week had average O3i values < 4%. Regarding supplementation, 12 of 60 (20%) cadets reported taking an omega-3 or fish oil supplement. On average, cadets that did not supplement had an O3i < 4%, whereas those that supplemented had an O3i > 4%.

### Erythrocyte Fatty Acid Status

**Figure 2.**
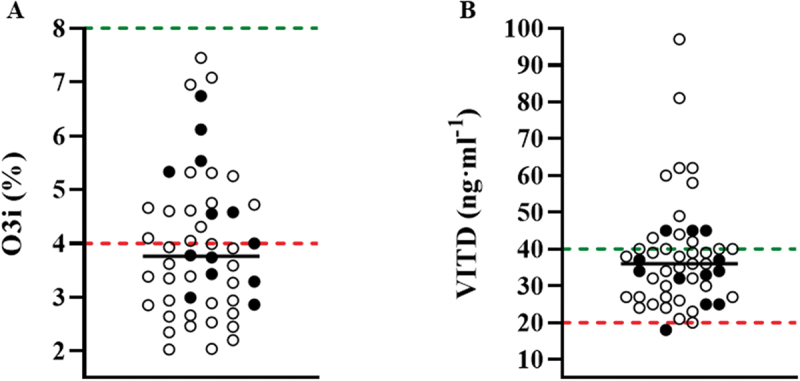
Individual data points (n = 50) for A) the omega-3 index (O3i) and B) serum vitamin D (VITD). The black line denotes the median values (O3i, 3.76%; VITD, 36.38 ng·ml^−1^), whereas the values above the green and below the red dashed line represents the optimal and suboptimal ranges, respectively. ● = female cadets (n = 13); o = male cadets (n = 37).

Figure 2 shows O3i for each cadet. Most cadets had an O3i in the sub-optimal zone (< 4%, [58%, *n* = 29]). EPA and DHA were 0.48% ± 0.33 and 3.43% ± 1.13, respectively.

As indicated in Table 4, the O3i and DHA was not associated with any ACFT performance outcome. Erythrocyte EPA was positively and moderately correlated with 3DL (*p* = .049), SPT (*p* = .027), LTK (*p* = .047), and strongly correlated with the PLK (*p* = .003). Based on the hierarchical multiple linear regression analysis, erythrocyte EPA (%) predicted PLK (*p* < .006) scores after controlling for body composition. After accounting for LBM, EPA was no longer predictive of 3DL (*p* = .347), SPT (*p* = .186), or LTK (*p* = .080) performance. Erythrocyte EPA accounted for 66.4% of the variance in the PLK scores. Every 0.1% increase in EPA translated into 5.4 (95% CI: 2.1, 8.8) better PLK scores.

**Table 4.** Correlations table for the relationship between ACFT performance, LC n-3 PUFAs, and VITD.

Performance Scores	O3i	EPA	DHA	VITD
3-RM Deadlift	0.104	0.280*	−0.013	0.305*
Standing Power Throw	0.171	0.314*	0.039	0.271
Hand-Release Pushup	0.028	0.146	0.002	0.355*
Sprint-Drag-Carry	−0.169	0.115	−0.215	0.260
Leg Tuck	0.280	0.316*	0.182	0.297
Plank	0.210	0.837†	0.039	−0.575
2 Mile Run	−0.146	−0.019	−0.115	0.326*
ACFT Score	0.035	0.230	−0.041	0.359*

Abbreviations: ACFT, Army Combat Fitness Test; LC n-3 PUFA, long-chain omega-3 polyunsaturated fatty acids; VITD, vitamin D; RM, repetition maximum

Note: *p < .05, †*p* < .01

### Vitamin D Status

Serum VITD status for each cadet is noted on Figure 2. Two cadets (4%) were VITD deficient (< 20 ng∙ml^−1^). VITD was insufficient (<30 ng∙ml^−1^) in 26% of cadets and below the optimal performance range (<40 ng∙ml^−1^) in 50% of cadets [31].

As noted in Table 4, serum VITD was positively and moderately correlated with 3DL (*p* = .031), HRPU (*p* = .011), 2MR (*p* = .021), and total ACFT score (*p* = .011). Using hierarchical multiple linear regression, serum VITD predicted 3DL (*p* = .002), HRPU (*p* = .012), 2MR (*p* = .032), and total ACFT (*p* < .001) scores after controlling for body composition (LBM and %BF). Serum VITD accounted for 9.3%, 12.6%, 8.7%, 11.0% of the variance in the 3DL, HRPU, 2MR, and total ACFT scores, respectively. Every 1 ng∙ml^−1^ increase in VITD was associated with a 0.3-point increase in each event. The overall ACFT score was associated with a 1.3-point increase for every 1 ng∙ml^−1^ increase in VITD.

## 4. Discussion

To our knowledge, this is the first study to examine the relationship between body composition, erythrocyte LC n-3 PUFAs, and serum VITD on ACFT performance outcomes. We hypothesized that LBM, LC n-3 PUFAs, and VITD would be significantly correlated with the ACFT, especially the strength and power events. In cadets, we demonstrated that body composition, %BF and LBM, were predictors of ACFT performance. Lower %BF predicted better performance on all ACFT events, except the PLK, whereas higher LBM predicted better performance on the 3DL, SPT, and SDC events. As expected, we found that FFMI_adj_ was linearly correlated with 3DL, SPT, HRPU, SDC, and overall ACFT scores. The recommended 15^th^ percentile FFMI_adj_ cutoff appears to identify those at risk of underperforming on the ACFT, and by extension, combat-related tasks. Cadets below the cutoff significantly underperformed on all ACFT events except for the PLK and had a higher rate of ACFT failures. Additionally, cadets reported a suboptimal dietary intake of LC n-3 PUFAs and had similarly low erythrocyte levels of EPA and DHA (average O3i < 4%). This may be a salient finding as EPA was positively associated with 3DL, SPT, LTK, and PLK, although it only remained strongly predictive of PLK scores after controlling for body composition. Serum VITD was positively correlated with 3DL, HRPU, 2MR, and overall ACFT scores. These relationships remained significant after adjusting for body composition.

While it is clear that %BF continues to be an important contributor to combat fitness, these results also suggest that LBM and other measures of muscularity, such as FFMI, are similarly critical to military and combat task performance, as measured by the ACFT. Our findings regarding the relationship between EPA and VITD provide insight into the potential nutritional interventions that may assist military personnel to achieve the physical demands associated with combat tasks.

### Body Composition

The Army’s recent transition to the ACFT may necessitate a reevaluation of the current Army body composition standards. The previous assessment, the APFT, focused on muscular and aerobic endurance, whereas the newly developed ACFT includes additional physical domains – strength, power, speed, and agility – associated with muscle mass. Although our hypothesis was related to LBM, we found that lower %BF was strongly correlated with higher scores on all ACFT events, except the PLK. Our findings are consistent with military studies that report lower %BF is associated with better 2MR [7,8] and push-up scores [8]. Despite the Army’s shift to the ACFT that is more focused on strength, power, speed, and agility; our investigation indicates that %BF has proven to be an important contributor to success on events that assess these physical domains, and therefore, performance of combat-relevant tasks. As expected, LBM contributed significantly to performance on the strength (3DL), power (SPT), and sustained anaerobic capacity (SDC) events. Previous studies have reported moderate to strong correlations between LBM and military relevant domains such as maximal lift capacity, anaerobic performance, and power output [5,35]. While LBM was correlated with HRPU scores, controlling for %BF attenuated the association between LBM and HRPU performance. Similarly, Farina et al. [8] found that lower %BF predicted better performance on the push-up event, whereas LBM did not.

Body composition analysis through the DXA allowed us to separate the components of FFM into LBM and BMC. However, military personnel use circumference-based equations that only capture FFM and BF, thus, FFMI may be the preferred method to assess muscularity. Similar to LBM, we found that FFMI was moderately correlated with HRPU and strongly correlated with 3DL, SPT, SDC, and overall ACFT scores. In agreement, a recent study using bioelectrical impedance analysis determined that FFMI were strongly correlated with ACFT scores [10]. While the investigation did not report performance outcomes for each event, FFMI is clearly an important variable for success on the ACFT. Harty et al. [9] proposed the use of lower-limit FFMI thresholds, the 15^th^ percentile, to identify military personnel in need of additional physical training and/or nutritional interventions aimed at increasing LBM. While our results generally agree, further investigations with much larger datasets are warranted to establish appropriate lower-limit FFMI thresholds.

### Long-Chain Omega-3 Polyunsaturated Fatty Acids

The role of LC n-3 PUFAs in cardiovascular health has been widely appreciated [36]. Recently, there has been an increased focus on the potential role of LC n-3 PUFAs on cognitive and physical performance in military and athlete populations [37–39]. Although low LC n-3 PUFA status does not manifest as a typical deficiency (e.g. vitamin C), it is clear that maximal incorporation of EPA and DHA into skeletal muscle phospholipid results in a range of physiological processes related to muscle protein synthesis and breakdown, modulation of inflammation, insulin signaling, and muscular recovery from physical training and injury [22,37]. In conjunction with the notable skeletal muscle applications [39], LC n-3 PUFA supplementation has also been shown to reduce biomarkers of head trauma (neurofilament-light) in athletes subjected to repetitive head impacts [40,41]. While this seminal publication only supplemented DHA [40], a recent study used a formula that included EPA and yielded similar reductions in neurofilament-light compared to a control group [41]. Despite this notable preliminary data, dietary intake and blood levels of LC n-3 PUFAs are consistently reported as suboptimal in military and athlete populations [12–15,18]. Cadets in the present study only consumed about ~12% of the daily recommend amount of 500 mg EPA+DHA [30] and, unsurprisingly, exhibited a suboptimal O3i (3.96%). More specifically, 58% of cadets (n = 29) had sub-optimal O3i (< 4%) status and no cadet met the criteria for optimal O3i status (> 8%). Our results are similar when compared to United States active duty military personnel. Previous investigations have reported O3i values of 3.45% and 3.88% in cohorts ranging from ~80 to ~400 soldiers [12,42].

We found that erythrocyte EPA, but not DHA, was positively correlated with the 3DL, SPT, LTK, and PLK events of the ACFT. However, after adjustment for body composition, the effect of EPA on 3DL, SPT, and LTK was attenuated, while the effect on PLK remained. Every 0.1% increase in EPA was associated with a substantially longer PLK hold of ~18 s. While the majority of studies exploring the relationship between LC n-3 PUFAs and performance tend to be in older populations, two recent investigations in young adults and athletes largely mirror the results noted in our study [15,24]. Using baseline maximal voluntary contraction of the elbow flexors, Ochi et al. [24] found that plasma EPA, but not DHA, was associated with strength in 32 young males. When divided into quartiles, subjects with the highest plasma levels of EPA were significantly stronger than those in the lowest quartile. In a recent cross-sectional analysis from our lab, we reported a significant association between whole blood EPA and handgrip strength in 36 college athletes [15]. EPA is strongly predictive of PLK performance and higher PLK scores will lead to higher overall ACFT scores. However, the PLK is likely the least combat relevant event within the ACFT and therefore may provide little useful information when Army leaders are looking to the ACFT to determine if their soldiers are combat ready. As such, it is unclear how much influence EPA will have on performance outcomes associated with combat tasks. To further elucidate the role of EPA on performance, supplementation trials should be conducted in various military relevant scenarios.

Combined with recent reviews on the potential physical and cognitive benefits associated with LC n-3 PUFA supplementation [38,39], our data adds to the relevant literature that LC n-3 PUFAs may play a role in the optimization of physical performance among military personnel. Additionally, recent data has illustrated the negative impact of military training, whether from the food environment or training itself, on LC n-3 PUFA status [18]. Hence, strategies should be in place to ensure the preservation of erythrocyte EPA and DHA status throughout the lifecycle of military service. In agreement, a recent military panel at the *Nutritional Armor for the Warfighter* conference unanimously stated that ‘it is unethical to do nothing’ to improve the n-3 PUFA status of military personnel [43]. While the study design limits our ability to imply causality, prudent strategies can be employed to maintain and improve LC n-3 PUFA status. Strategies include updating the food environments to encourage and provide more LC n-3 PUFA options and, potentially, less n-6 PUFAs; screening for EPA and DHA status during basic military training and periodic health assessments; and providing a standardized intervention for O3i < 4%.

### Vitamin D

Beyond the well-established effects on bone and calcium health, VITD participates in many extraskeletal physiological and pathological processes via the ubiquitous vitamin D receptor [44], including muscle protein turnover and skeletal muscle repair, recovery, and regeneration [21]. Unfortunately, recent studies have shown that military personnel exhibit high levels of VITD insufficiency with estimates ranging from 57% to 86.1% [12,45]. In our study, only 26% of cadets were VITD insufficient. While many factors may have contributed to this difference, the most plausible are sun exposure and the season of data collection. As described by Holick [44], minimal VITD is produced from sun exposure above 35° N latitude (Atlanta) and our study location was below this latitude (31.5° N). Since sun exposure varies throughout the year, our lower prevalence of VITD insufficiency may also be explained by the majority of our cohort being tested when serum VITD concentrations are typically at the highest (September – November).

The military has recognized the importance of VITD, namely, its role in the pathogenesis of stress fractures and the significant costs associated with treatment and recovery [46]. However, an underappreciated benefit related to VITD status may be its potential effects on physical performance. The present study found that serum VITD was positively associated with strength (3DL), muscular (HRPU) and cardiorespiratory endurance (2MR) events as well as overall ACFT scores. One study in military personnel reported similar muscular performance outcomes [47]. In 100 soldiers, Barringer et al. [47] reported a significant positive association with VITD status and push-up performance on the APFT. While this investigation did not adjust for body composition, our results for 3DL and HRPU scores remained significant after controlling for LBM. In fact, for every 10 ng∙ml^−1^ increase in VITD, 3DL and HRPU increased by approximately 5 kg and 3 repetitions, respectively (~3 points each). Meta-analyses of interventional studies have reported similar results in athletes [48,49]. Both meta-analyses reported that VITD supplementation improve lower-limb strength [48,49], while only Tomlinson et al. [49] reported an increase in upper-body strength. Of note, the latter meta-analysis included four studies in non-athlete populations. A more recent trial involving athletes, not included in the aforementioned meta-analyses, found that 1RM DL significantly improved following 12-weeks of seasonal VITD supplementation compared to the control group [17].

Our finding that VITD status may be related to cardiorespiratory fitness (2MR) has been shown previously [16]. After controlling for body composition, we found that for every 1 ng∙ml^−1^ increase in VITD, cadets performed 1.3 s faster on the 2MR. In a cross-sectional study including 967 military trainees, Carswell et al. [16] found that serum VITD was predictive of 1.5 mile run time. After adjusting for body composition and other factors, every 1 ng∙ml^−1^ increase in VITD was associated with ~0.5 s faster on the run event. Taken together, these findings are practically relevant, as a 20 ng∙ml^−1^ increase in VITD would amount to a 26s and 10s improvement in the 2MR and 1.5MR, respectively.

While it is well established that suboptimal VITD status may have a profound effect on bone health, our results imply that VITD may play a pivotal role in military performance optimization, especially in relation to sustained and improved combat readiness. Recent commentaries [46] and editorials [50] within the military sphere have highlighted the need for a greater focus on preventive, cost-effective, and pragmatic approaches to ensure military personnel reap the benefits associated with adequate VITD status, especially as a stress fracture risk mitigation strategy. While the data here cannot be prescriptive, the recommended strategies to improve Warfighter VITD status, proposed by Fogleman and colleagues, are prudent and appear to be in-line with the current state of the evidence [46]. These recommendations include screening of military recruits entering service, daily sun exposure (at least 20 minutes), consumption of VITD-rich and fortified foods, routine seasonal supplementation, and standardized treatment protocols for low VITD.

### Strengths and Limitations

This study was the first to explore the relationships between body composition, LC n-3 PUFA, VITD status and ACFT performance. One limitation of this preliminary research is the modest sample size. While the study design employed may limit our ability to imply causality, our observations regarding biomarkers and ACFT performance are strengthened by adjusting for body composition. Although physical activity data was not obtained, the cadets were required to conduct group physical training four days per week. This may limit our interpretation; however, we speculate that the similar physical training reduces the individual variation in exercise regimes and, thus, may not substantially influence our reported performance outcomes. Similarly, we were unable to account for other dietary variables that may influence performance (e.g. carbohydrate, protein, etc); however, previous evidence has shown that military personnel have sub-optimal intake of omega-3 and VITD, not necessarily total macronutrient intake. Of note, we used a strict standardized assessment protocol and one of the most reliable methods for body composition analysis (DXA).

In summary, body composition, namely %BF and LBM or FFMI, are important contributors to ACFT performance. Since measures of muscle mass (LBM, FFMI) are associated with important performance metrics on the ACFT, the Army body composition standards may need to be reevaluated. Large military datasets should be utilized to establish lower-limit FFMI thresholds. As previously described, the optimization of LC n-3 PUFAs and VITD status within military populations has important health, performance, and cognitive applications [39,51]. As a proxy for combat readiness, the beneficial effects on ACFT performance associated with optimal EPA and VITD status in the present study illustrates the potential clinical utility of monitoring these biomarkers upon entry and throughout military service. Since body composition, EPA, and VITD status are all modifiable, practitioners can use this knowledge to provide military personnel with exercise and nutrition guidance to improve ACFT performance. Future intervention studies should focus on these variables in various military scenarios.
